# Peculiar elastic behavior of mechanical metamaterials with various minimal surfaces

**DOI:** 10.1038/s41598-019-38660-1

**Published:** 2019-02-27

**Authors:** Jun-Hyoung Park, Jae-Chul Lee

**Affiliations:** Department of Materials Science and Engineering, Korea University, Seoul, 02841 South Korea

## Abstract

Molecular dynamics simulations were performed on nanostructured metamaterials (NMs) with gyroid, diamond, and primitive structures to evaluate their mechanical behavior, especially elastic properties. Unlike the constant nature of Young’s (*E*) and shear (μ) moduli of bulk materials, the values of both *E* and μ of NMs change with relative density and cell size but at different rates depending on the morphologies of the structure. This is particularly the case for μ; for a given relative density and cell size of NMs, the μ values differ greatly, depending on the types of structure, causing the NMs to display differing μ/*E* values and thus resistance to shear deformation. The mechanistic origin of this observation was analyzed by resolving the morphologies of the NMs in terms of the numbers and orientations of the fundamental structural motifs for constructing metamaterials.

## Introduction

Several different nanostructures with various types of minimal surfaces have been found in living organisms^1–7^. Minimal-surface structures are characterized by triply bicontinuous isotropic structures and display unusual/unique optical^8–10^, electromagnetic^11,12^, and hydrodynamic properties^13,14^. Extensive studies to reveal the physics underlying their unique properties have been conducted by replicating two-dimensional (2D) nanoscale structures using photolithography and selective etching techniques^15,16^. The results commonly state that the properties of these nanostructured metamaterials (NMs) are predominantly determined by their microscopic morphologies rather than chemical composition^8,17^. In contrast to the extensive literature on 2D metamaterials, studies on the mechanical properties of three-dimensional (3D) metamaterials are relatively scarce because of the difficulty and complexity in analyzing their morphologies.

With the development of computational tools and manufacturing technologies of nanostructures, studies on 3D metamaterials are underway to establish their structure-property relationship^17–19^. Lee *et al*., according to their finite element method (FEM) analyses on the elastic properties of various metamaterials, found that the relative density and aspect ratio of unit cells play a major role in determining their elastic properties^20^. Kang *et al*. measured the stress–strain responses of μm-scale metamaterials and reported that the relative thickness of the strut is an important parameter affecting the elastic properties of metamaterials^21^. Although earlier studies were instructive for evaluating the unique elastic properties of metamaterials, these analyses were conducted for metamaterials with predetermined/particular configurations and did not explain properly why they behave so. More importantly, FEM is a large-scale analysis tool and thus does not consider the effect arising from the surface energy of *nanoscale* structures, which can be found from nature. This indicates that any description based only on FEM is insufficient for explaining the structure-property relationship of “nanoscale” structures, especially those with small relative densities. From this perspective, to properly interpret the mechanistic origins responsible for unique mechanical behaviors of NMs, an appropriate method is necessary that simultaneously considers both the surface effect and structural configurations of NMs.

All metamaterials with minimal surfaces, although seemingly complex, are made of fundamental substructures i.e., interconnected beams (referred to as “struts”) and their connection points (referred to as “nodes”). This renders nanostructured metamaterials to display three distinctive structural features. First, owing to their porous nature, the elastic properties of all NMs are affected by their relative density (ρ) (i.e., the ratio of the volume enclosed by the minimal surface to that of the unit cell)^20,21^. Second, because metamaterials are the supercell structures constructed by repeating unit cells with a given cell size (*L*), their mechanical properties are influenced by the *L* value^17^. Finally, the morphologies of metamaterials are determined by how struts are connected to a node. Therefore, the population and orientation of struts aligned along the direction of loading are the factors that most significantly affect their mechanical properties. While previous studies reported the effect of the morphologies characterized by the *ρ* and *L* values of metamaterials on their mechanical properties, these studies did not explain the resultant properties from the perspective of the fundamental structural motifs of the metamaterials.

In this study, comparative investigation was performed on various NMs using molecular dynamics (MD) simulations to elucidate the structural origin of the mechanical properties of NMs. For this purpose, we prepared three representative nanostructured metamaterials with single gyroid (G), single diamond (D), and primitive (P) structures (hereinafter, denoted as G-, D-, and P-NMs, respectively). MD simulations of the NMs showed that, unlike the constant nature of elastic coefficients of isotropic bulk materials, Young’s (*E*) and shear (μ) moduli of the NMs change with the values of *ρ* and *L* but at different rates depending on the types of the NMs. This observation was analyzed by resolving the morphologies of the model NMs in terms of the numbers and orientations of the fundamental structural motifs, i.e., the strut and node, of the metamaterials.

## Results and Discussion

### Preparation of NMs

**Figure 1:**
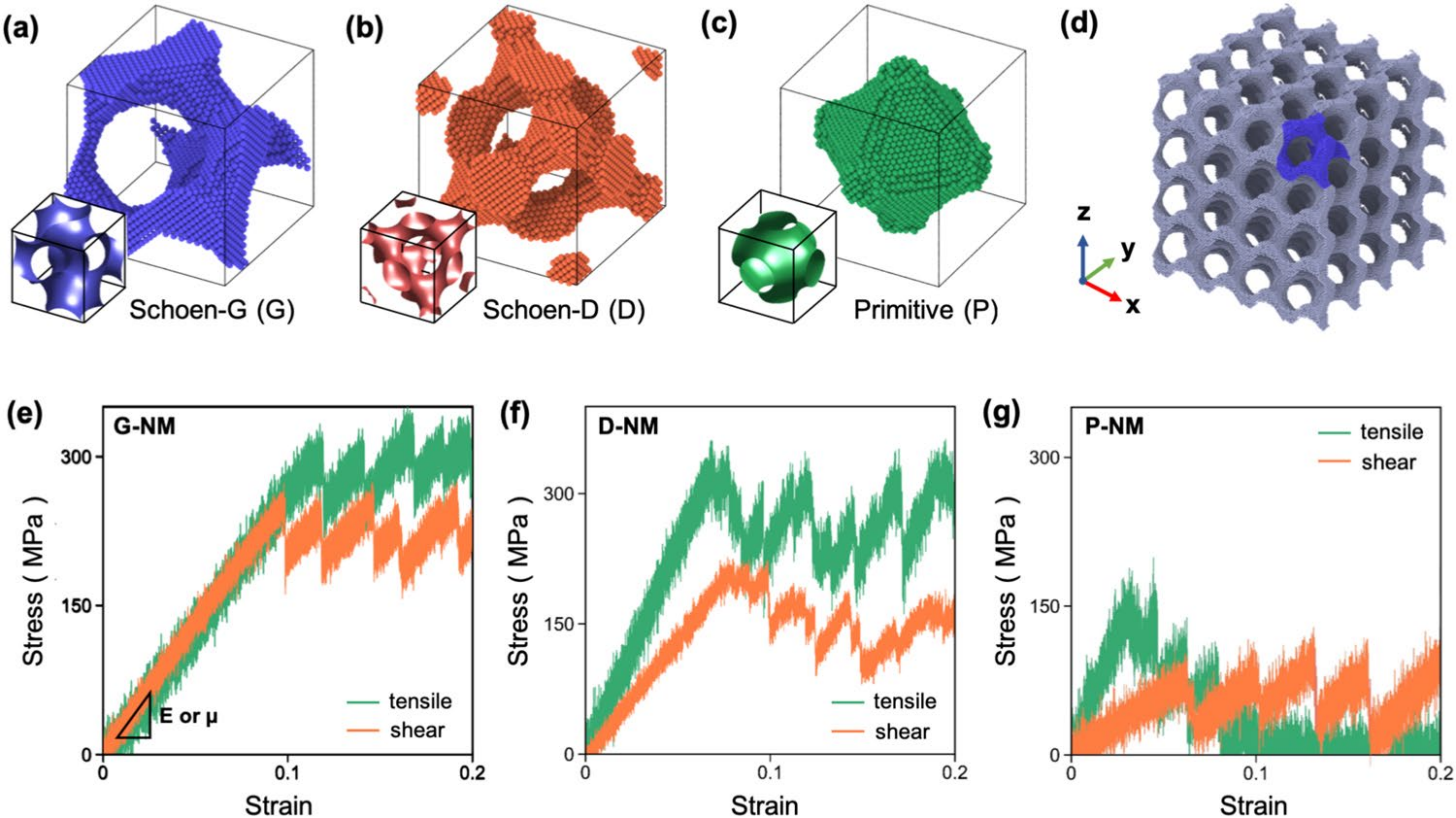
Unit cells of the metamaterials with (**a**) G, (**b**) D, and (**c**) P structures. The insets of (**a**), (**b**), and (**c**) correspond to the level surfaces (also called the minimal surfaces) of each model structure. (**d**) Example showing the G-NM constructed by applying the periodic boundary conditions. The stress–strain curves of the (**e**) G-, (**f**) D-, and (**g**) P-NMs (*L* = 8 nm and *ρ* = 0.25) obtained from MD simulations under uniaxial tension (denoted in green) and simple shear (denoted in orange).

To model the NMs and evaluate their elastic properties, we first generated the minimal surfaces (also termed the level surfaces) corresponding to the G, D, and P structures using the following equations^22,23^:

1 $${S}_{x}^{1}{C}_{y}^{1}+{S}_{y}^{1}{C}_{z}^{1}+{S}_{z}^{1}{C}_{x}^{1}=C\,{\rm{for}}\,{\rm{Schoen}}\mbox{--}{\rm{G}}$$

2 $${C}_{x}^{1}{C}_{y}^{1}{C}_{z}^{1}+{C}_{x}^{1}{S}_{y}^{1}{S}_{z}^{1}+{S}_{x}^{1}{C}_{y}^{1}{S}_{z}^{1}+{S}_{x}^{1}{S}_{y}^{1}{C}_{z}^{1}=C\,{\rm{for}}\,{\rm{Schoen}}\mbox{--}{\rm{D}}$$

3 $$-({C}_{x}^{1}+{C}_{y}^{1}+{C}_{z}^{1})=C\,{\rm{for}}\,{\rm{P}}$$

where ${S}_{\alpha }^{n}=\,\sin (2n\pi \frac{\alpha }{L})$ and ${C}_{\alpha }^{n}=\,\cos (2n\pi \frac{\alpha }{L})$, where *L* is the unit cell size, and *C* is the threshold of the level surface that determines the relative density (or the volume fraction, *ρ*). NMs with differing structures were produced by eliminating atoms that did not satisfy Eqs (1–3) from the single crystalline Al cube with its <001> direction parallel to the tensile direction (i.e., the *z*-axis). The unit-cell structures of the metamaterials along with their corresponding minimal surfaces are shown in Fig. 1a–c. Periodic boundary conditions were then imposed along the *x*-, *y*-, and *z*-axes to prepare the supercell structures of the metamaterials for mechanical tests (e.g., Fig. 1d). It is noted that the designed metamaterials are anisotropic because they are based on the single crystalline Al. However, the degree of anisotropy (or the Zener ratio = 2C_44_/(C_11_ − C_12_) in Voigt notation) is particularly small (=1.22) for single-crystalline Al, which is still close to the isotropic value of 1. Therefore, the crystallographic orientation effect can be ignored for the whole cell.

### Evaluation of mechanical properties

Fig. 1e–g show the examples of the stress–strain curves measured from the G-, D-, and P-NMs with *L* = 8 nm and *ρ* = 0.25 (detailed values of elastic/plastic properties are listed in Table 1). All NMs are characterized by high yield strength and serrated plastic flow during deformation. However, unlike the serrated flows observed from various work-hardenable alloys, such as Al-Mg alloys^24^, bulk amorphous alloys^25^, twin-induced plasticity steel^26^, the plastic flow of the NMs proceeds in the absence of work hardening. This hardening behavior is typical of most nanostructured materials, because they are unable to accommodate generated dislocations owing to their small dimensions. However, the plastic deformation issue of NMs is not relevant to the scope of the present study and thus will not be discussed further. In the following, we focus our attention on the elastic properties of the NMs.

**Table 1:** Values of yield strength (σ_*y*_ and τ_*y*_), elastic limit (ε and γ), Young’s modulus (*E*), shear modulus (μ), and Poisson’s ratio (ν) evaluated for various NMs with *L* = 8 nm and *ρ* = 0.25.

Structure		G	D	P
Yield strength (MPa)	σ_*y*_	296	325	137
	τ_*y*_	244	203	68
Elastic limit (%)	ε	12	6.8	3.0
	γ	9.8	7.4	6.0
Young’s modulus, E (GPa)		2.6	4.7	5.0
Shear modulus, μ (GPa)		2.6	3.1	1.2
Poisson’s ratio, ν		0.37	0.38	0.05
μ/E		1.0	0.66	0.24

Note that every elastic property was obtained from the elastic region with strains less than 3%.

It is noted from the stress–strain curves (Fig. 1e–g) that the elastic limits of the metamaterials (except for the P-NM) are ~10%, which is much greater than the elastic limits of polycrystalline bulk metals (<0.3%)^24,27^, perfect crystalline metals (~5%)^28^, and amorphous alloys (2–3%)^28^. Of particular interest is that the values of *E* of the NMs are similar, whereas μ differs significantly depending on the type of metamaterial. These characteristics caused the NMs to display different μ to *E* ratios (hereinafter, referred to as the μ/*E* value). For example, for the NMs with *L* = 8 nm and *ρ* = 0.25, the μ/*E* value of G-NM is 1.0, whereas that of P-NM is only 0.24. These values differ largely from that (~0.38) of most isotropic bulk metals. Because *E* and μ are the structural parameters that can measure the resistance to tensile and shear deformation, respectively, the μ/*E* value can be used as a descriptor that can assess the load-carrying capability of NMs under shear deformation.

### Elastic moduli of various NMs

**Figure 2:**
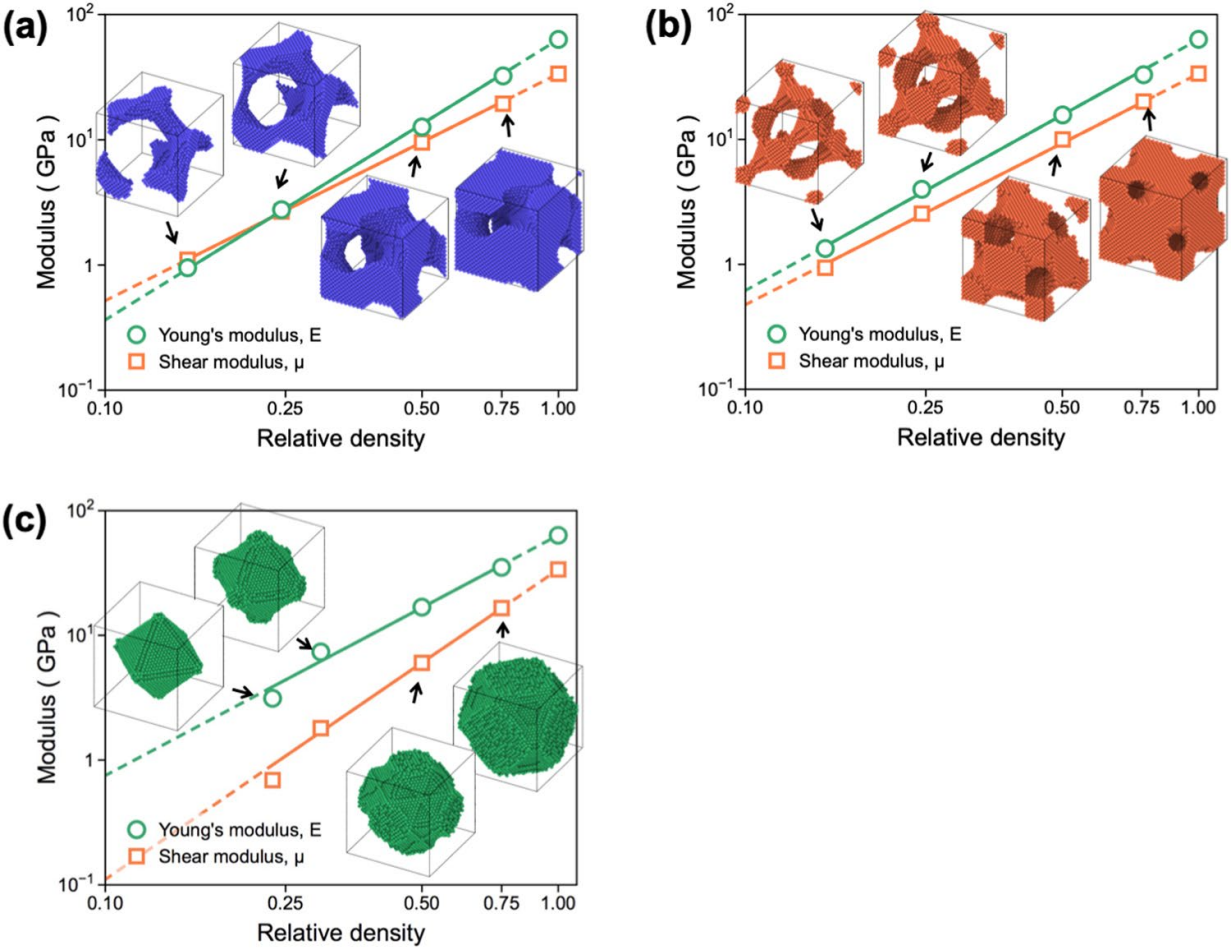
Changes in the values of *E* and μ evaluated as a function of the relative density of the NMs with (**a**) G, (**b**) D, and (**c**) P structures. Note that the insets in the graph are the morphologies of the unit cell (*L* = 8 nm) constructing the supercell structures of G-, D-, and P-NMs used to determine the mechanical properties.

Before analyzing the mechanistic origin of different μ/E values displayed by the NMs with differing morphologies, we first evaluated the elastic properties of the NMs as a function of the relative density (*ρ*) and cell size (*L*). Fig. 2a–c show the changes in the values of *E* and μ evaluated as a function of *ρ* for the G-, D-, and P-NMs with *L* = 8 nm. Although the values of both *E* and μ naturally decrease with decreasing *ρ*, the decreasing rates of the strength and modulus of each NM differ significantly depending on the types/morphologies of the NMs. The *E* values and their decreasing rates are less sensitive to the types of NMs considered in this study. On the other hand, the μ values differ significantly depending on the types of metamaterials, such that the decreasing rate of μ is faster in the order of G, D, and P structures. Notably, this different decreasing tendency is not observed in previous FEM-based studies because this behavior displayed by differing NMs are attributed not only to the morphology but also to the cell size and relative density (equivalent to surface energy) of the NMs.

### Surface effect on μ/E values

**Figure 3:**
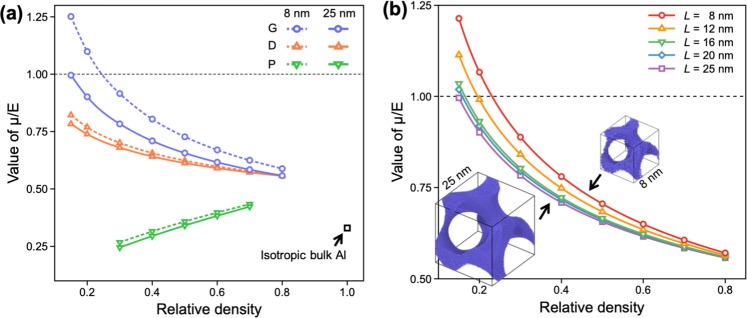
(**a**) Variations in the values of μ/*E* evaluated as a function of the relative density of the NMs with G, D, and P structures and cell size. Each NM has a different mechanical anisotropy depending on the relative density, cell size, and morphology. (**b**) Changes in the values of μ/*E* evaluated as a function of the relative density of the G-NM with various cell sizes.

Figure 3a shows the variations in the μ/E values of the G-, D-, and P-NMs evaluated over a wide range of the relative density (*ρ* = 0.15–0.75) and unit cell size (*L* = 8–25 nm). It is noted that the μ/E values of the NMs considered in this study tend to converge to 0.5–0.6 regardless of the cell size, as the *ρ* value approaches a possible maximal value (~0.8) needed to construct the triply bicontinuous structures^29^. However, as *ρ* decreases, the μ/*E* values of the NMs diverge; for *ρ* = 0.3, the μ/*E* values of the G- and D-NMs increase gradually from 0.53 to values greater than 0.70, whereas the μ/*E* value of the P-NM decreases to 0.25. In addition to *ρ* of the metamaterials, *L* is another parameter that influences the elastic properties of the NMs. For example, as *L* of the gyroid unit cell increases from 8 to 25 nm, the μ/*E* values of G-NMs decrease gradually and converge to saturation beyond *L* = 25 nm, as seen in Fig. 3b. It is noted that the μ/*E* value of the G-NM with *L* = 25 nm is very similar to those evaluated for the large-scale structure constructed using finite element analyses^20^. This tendency was also observed for D- and P-NMs (see the solid lines in Fig. 3a), indicating that any structures with *L* >25 nm exhibit elastic properties similar to those of large-scale metamaterials.

Referring back to Fig. 3a, even for the structures with the same *L* and *ρ* values (thereby, the similar surface energy), the μ/*E* values of the NMs differ largely depending on their morphologies, indicating the existence of additional factors responsible for the unique elastic properties of the metamaterials. It is noted that, in addition to the *L* and *ρ* values, the structures of NMs are also characterized by their distinct morphologies (i.e., the trigonometric terms in Eqs (1–3)) of the unit cell. Therefore, to elucidate the mechanistic origins responsible for the changes in the elastic properties of the NMs, it is necessary to analyze the structure–property relationship by resolving the structures of the NMs into fundamental substructures determining their morphologies, while considering the surface effect.

### Tensile behavior of NMs

**Figure 4:**
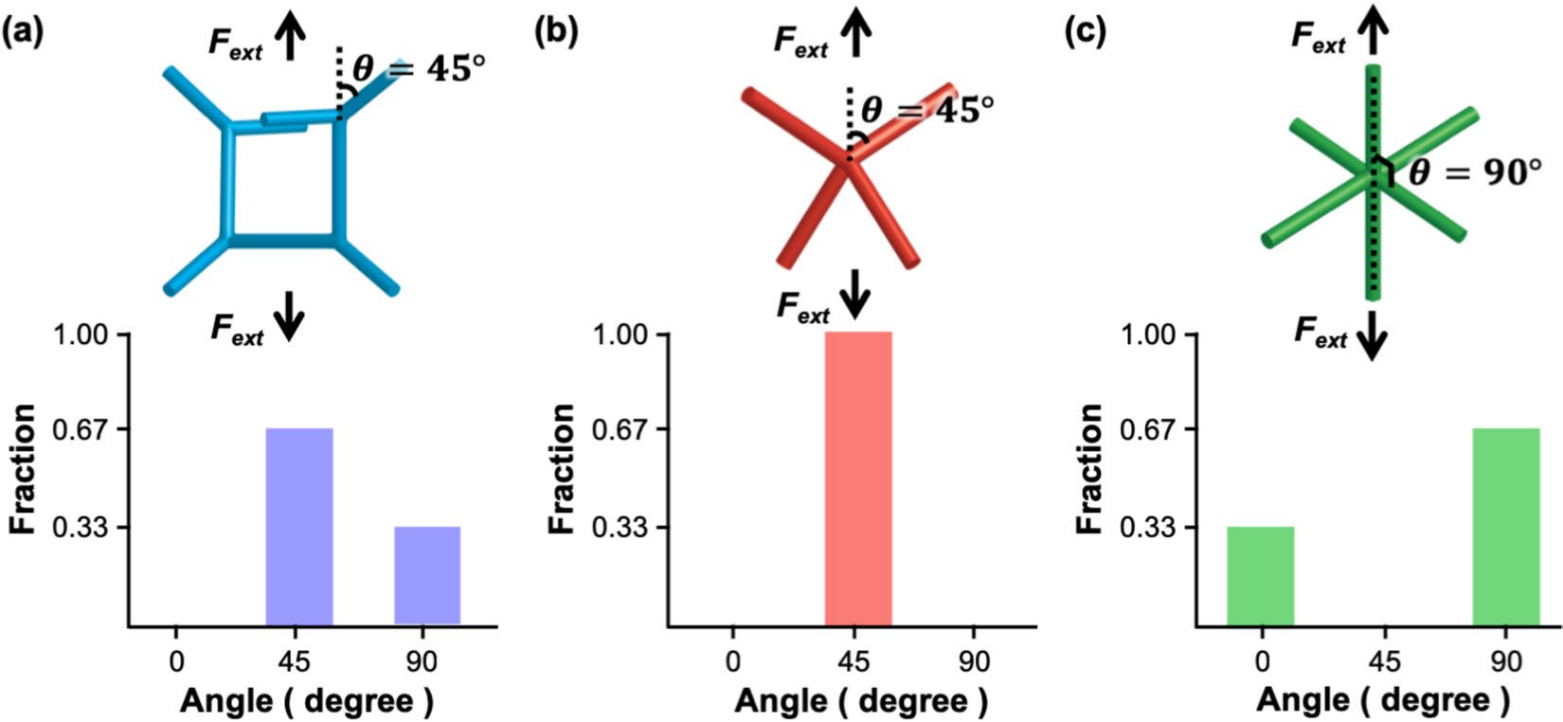
Fractions of the struts with the angle (*θ*) with respect to the loading direction: (**a**) G, (**b**) D, and (**C**) P structures. The schematics in the graphs are the nonvolumetric framework representations of the unit cell of the metamaterials.

Every metamaterial with minimal surfaces, while seemingly complicated, is made of the fundamental structural motifs, i.e., the strut and node. Because struts are the structural motifs that can sustain the majority of the load imposed on NMs, the mechanical properties of NMs rely on the number and orientation of the struts. For each metamaterial, the total numbers of struts are similar regardless of the type of metamaterial. However, the numbers of struts connected to a node differ depending on the type of metamaterial; these values are three, four, and six for G-, D-, and P-NMs, respectively (see the schematics in the insets of Fig. 4). This causes the connection angle of the struts to the node to vary depending on the type of metamaterial. When an external load is applied to these materials, the orientations (*θ*) of struts relative to the loading direction differ depending on the structures, which in turn determines the mechanical properties of metamaterials. With this in mind, we resolved the structure of NMs into the strut and node and analyzed their effect on the mechanical behavior according to their orientations with respect to the loading direction.

When analyzing the types of struts according to the *θ* values, the struts are classified by those aligned along *θ* = 0, 45, and 90°. Struts with different *θ* values would exhibit differing load carrying capability and deformation characteristics. For example, struts with *θ* = 0° can withstand the highest tensile load with their deformation governed only by stretching, whereas struts with *θ* = 45° carry the tensile load by the simultaneous action of stretching and rotation. On the other hand, struts with *θ* = 90° are unable to sustain the tensile load and thus are nearly free from deformation. Therefore, the capacity of the NMs for carrying the tensile load is determined by the numbers of struts with *θ* = 0 and/or 45° constructing the NMs. Fig. 4a–c show the relative fractions of struts with *θ* = 0, 45, and 90° constructing the metamaterials. The fractions of struts with *θ* = 0 and 45° are small in the order of D, G, and P structures, indicating that metamaterials can withstand the tensile load in the same order. This explains why D-NM displays the highest yield strength (325 MPa), whereas P-NM exhibits the lowest strength (137 MPa), as shown in Fig. 1.

It was observed in Fig. 2a–c that the values of both *E* and μ differ greatly depending on the types of the NMs. For example, for three NMs with *L* = 8 nm and *ρ* = 0.30, the difference in the *E* values of the NMs is ~20%, whereas that in the μ values is ~220%. The comparatively small difference in the *E* values displayed by the NMs can be qualitatively explained by simultaneously considering the ability of the struts to carry the force (*F*_*ext*_) and associated displacement/elongation (Δ*L*) as per the relationship $E\propto {F}_{ext}/{\rm{\Delta }}L$. In the case of struts with *θ* = 45°, they can sustain the tensile load by the combination of stretching and rotation. Therefore, despite its high strength, D-NM, which is composed only of struts with *θ* = 45°, also suffers from a large deformation owing to the combined effect of the rotation and stretch of the struts. On the other hand, P-NM contain a small fraction of struts with *θ* = 0° and thus has the least capacity to carry the tensile load, as revealed by its lowest strength. However, this structure, when viewed from the perspective of deformation, is less willing to stretch under uniaxial tension because struts composing the structure are aligned along *θ* = 0°. This causes P-NM to display an *E* value comparable to those of the G- and D-NMs. In summary, the interplay of the load-carrying capability and resistance to tensile deformation of differing struts constructing NMs compensates for the individual effects that *F*_*ext*_ and Δ*L* have on the *E* values, explaining why the *E* value is less sensitive to the types of NMs. Therefore, a large difference in the values of μ/*E* observed from the NMs is considered to arise predominantly from the deformation characteristics of the NMs under shear loading.

### Shear behavior of NMs

**Figure 5:**
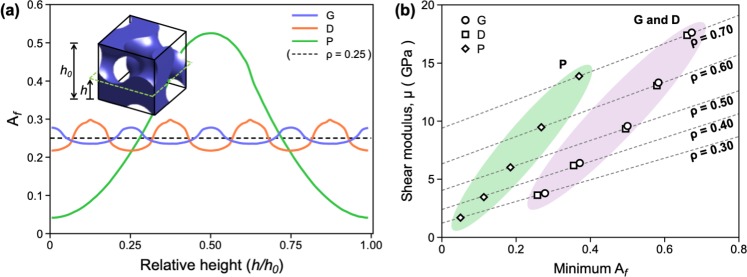
(**a**) Changes in the values of A_*f*_ evaluated as a function of the relative height (*h/h*_*o*_) in the *z*-direction. The inset is an example showing the relative height (*h/h*_*o*_) defined in the unit cell of the G structure. The dash line in the graph is the average A_*f*_ of the metamaterials with *ρ* = 0.25. (**b**) Changes in the values of μ measured as a function of the minimal A_*f*_ evaluated for the various NMs with differing *ρ* values.

Unlike tensile deformation, shear deformation proceeds by the sliding of one atomic layer over the neighboring layer. Therefore, for NMs with a given *ρ*, the load-carrying capability under shear load is proportional to the cross-sectional area of the loading plane, i.e., the plane parallel to the shearing direction. The cross-sectional area of NMs can be evaluated by the area fraction (A_*f*_) occupied by atoms on the loading plane of the unit cell, which varies along the relative height (*h/h*_*o*_) of the unit cell of the NMs. Fig. 5a shows the changes in the values of A_*f*_ measured as a function of the relative height of each NM. Of all NMs considered in this study, G-NM exhibits the most uniform distribution of A_*f*_, whereas P-NM exhibits the largest fluctuation in the values of A_*f*_. When a shear load is applied to these NMs, the shear stress is concentrated at the loading plane with the minimal A_*f*_. This makes the plane with the minimal A_*f*_ the facile site for shear deformation. Therefore, for a given value of ρ, the μ value would be linearly related to the value of the minimal A_*f*_ of the NMs.

Figure 5b shows the changes of the μ value evaluated as a function of the minimal A_*f*_ of the NMs for various ρ values. For a given *ρ* value of the structures, the μ value of the NMs decreases linearly in proportion to the minimal values of A_*f*_ in the order of G-, D-, and P-NMs. Therefore, compared to the G- and D-NMs, P-NM with the smallest minimal A_*f*_ is less resistant to shear deformation. This explains why P-NM displays the lowest μ and thus the smallest μ/*E* values, whereas G-NM, which exhibits the largest minimal A_*f*_, displays the largest μ and thus the highest μ/*E* values.

In conclusion, MD simulations performed on the NMs showed that values of both *E* and μ decrease with decreasing relative density, whereas the rates of decrease of *E* and μ differ depending on the type of structure; changes in the *E* value are comparatively less sensitive to the type of structure, whereas the changes in the μ value is structure-sensitive, rendering the μ/*E* values larger in the order of G-, D-, and P-structures. The differing μ/*E* values displayed by the NMs with the same values of *L* and ρ indicate that, in addition to the surface energy, the orientation of the struts constructing the morphologies of NMs is another important factor determining the mechanical behaviors of NMs; of the three NMs, D-NM has the most load-carrying capable struts (*θ* = 0 and 45°), causing it to display the largest tensile strength. On the other hand, P-NM contains the fewest load-carrying struts, exhibiting the smallest tensile strength. However, owing to the interplay of the load-carrying capability and resistance to tensile deformation of differing struts, *E* of the NMs is less sensitive to their types. Unlike tensile characteristics, the load-carrying capability under shear deformation is related to the minimal A_*f*_ of the unit cell constructing the NMs. For a given *ρ* value of the structures, the μ value of the NMs decreases linearly in proportion to the minimal values of A_*f*_ in the order of G-, D-, and P-NMs, which makes P-NM less resistant to shear deformation. This explains why P-NM displays the lowest μ and thus the lowest μ/*E* values, whereas G-NM displays the highest μ/*E* value.

## Methods

Various NMs with differing *L* and *ρ* were computationally generated by changing the values of *L* and *C* in Eqs (1–3) using conventional MD. To describe the interatomic interactions of Al atoms constructing the NMs, the embedded atom method (EAM) potential^30^ was employed in a large-scale atomic/molecular massively parallel simulator (LAMMPS)^31^. Prior to mechanical tests of the NMs, each structure was pre-relaxed using conjugate gradient energy minimization and subsequently equilibrated using a Nosé–Hoover thermostat at 300 K for 5 ns. Periodic boundary conditions were then imposed along the *x*-, *y*-, and *z*-axes to prepare the supercell structures of the metamaterials for tensile and shear tests.

To study the effect of the structural parameters on the values of *E* and μ of the NM, we calculated the stress–strain curves over a wide range of unit cell size (*L* = 8–25 nm) and relative densities (*ρ* = 0.15–0.75) with various morphologies (G, D, and P structures). Tensile tests were performed by applying uniaxial tension in the *z*-direction, whereas shear tests were conducted by applying shear load on the *xy*-plane (see Fig. 1d). A uniform strain loading condition (at the strain rate of 10^7^ s^−1^) was maintained during both tensile and shear tests of the NMs to avoid the shock wave loading effect. This was achieved by linearly varying the velocity of the individual atoms along the loading direction from zero at the bottom end to a maximal value (corresponding to ε and γ = 0.03) at the loading top end. The values of *E* and μ of the NMs were obtained from the slopes of the elastic region with strains less than 3% in the stress–strain curves measured during testing of the NMs (see Fig. 1e–g).
